# Alteration of Intestinal Microbiota Composition in Oral Sensitized C3H/HeJ Mice Is Associated With Changes in Dendritic Cells and T Cells in Mesenteric Lymph Nodes

**DOI:** 10.3389/fimmu.2021.631494

**Published:** 2021-06-10

**Authors:** Cui Zhou, Ling-Ling Chen, Rui-Qi Lu, Wei-Wei Ma, Rong Xiao

**Affiliations:** ^1^ Beijing Key Laboratory of Environmental Toxicology, School of Public Health, Capital Medical University, Beijing, China; ^2^ Nutritional Department, Handan First Hospital, Handan, China; ^3^ School of Basic Medicine, Capital Medical University, Beijing, China

**Keywords:** OVA sensitization, 16S rRNA, intestinal microbiota, dendritic cells, regulation T cells, *Clostridiales*, *Mollicutes_RF39*

## Abstract

This research aimed to investigate the allergic reaction of C3H/HeJ mice after sensitization with ovalbumin (OVA) without any adjuvant and to analyze the association between intestinal microbiota and allergy-related immune cells in mesenteric lymph nodes (MLN). The allergic responses of C3H/HeJ mice orally sensitized with OVA were evaluated, and immune cell subsets in spleen and MLN and cytokines were also detected. The intestinal bacterial community structure was analyzed, followed by Spearman correlation analysis between changed gut microbiota species and allergic parameters. Sensitization induced a noticeable allergic response to the gavage of OVA without adjuvant. Increased levels of Th2, IL-4, CD103^+^CD86^+^ DC, and MHCII^+^CD86^+^ DC and decreased levels of Th1, Treg, IFN-γ, TGF-β1, and CD11C^+^CD103^+^ DC were observed in allergic mice. Furthermore, families of *Lachnospiraceae*, *Clostridiaceae_1*, *Ruminococcaceae*, and *peprostreptococcaceae*, all of which belonging to the order *Clostridiales*, were positively related to Treg and CD11C^+^CD103^+^ DC, while they were negatively related to an allergic reaction, levels of Th2, CD103^+^CD86^+^ DC, and MHCII^+^CD86^+^ DC in MLN. The family of *norank_o_Mollicutes_RF39* belonging to the order *Mollicutes_RF39* was similarly correlated with allergic reaction and immune cells in MLN of mice. To sum up, allergic reactions and intestinal flora disturbances could be induced by OVA oral administration alone. The orders of *Clostridiales* and *Mollicutes_RF39* in intestinal flora are positively correlated with levels of Treg and CD11C^+^CD103^+^ DC in MLN of mice.

## Introduction

It is undisputed that food allergy (FA) has become one of the most important public health problems due to its remarkably increased prevalence worldwide in recent years (1). In the USA, the reported prevalence of food allergies was 7.6% in children (2) and 10.8% in adults (3). Although the prevalence of FA is not accurately known in China, the latest epidemiological survey in Wenzhou, China, reported a prevalence rate of 12.86% in preschool children (4).

As antigen-presenting cells (APCs), dendritic cells (DCs) in local immune tissue [peyer’s patch, mesenteric lymph node (MLN) and so on] (5) and peripheral immune organs (e.g., spleen) (6) play pivotal roles in the induction of tolerance and food allergy (7). It has been demonstrated that CD103^+^ DCs in MLN can promote the development of T regulatory cells (Tregs) (5), which can suppress Th2 response, thus, preventing FA and maintaining tolerance (8, 9). Therefore, the association among DCs, Tregs, and Th2 will influence the dynamic balance between T-helper type 1 (Th1) and Th2 cells in food allergic conditions.

Genetic variation, thought to be a vital factor inducing food allergy for many years (10, 11), could not, however, explain the dramatic increase in its prevalence (12–15). Therefore, researchers have proposed the hypothesis that interaction between the environment and the immune system may have contributed to allergic diseases because of changes in sanitary conditions, dietary habits, and surroundings (16), which are often accompanied by alteration in the symbiotic microbiota (11). The focus of research about food allergy has thus shifted toward commensal microbiota in recent years (16, 17).

The intestinal microbiota is found to be a key environmental factor in promoting oral tolerance (18, 19). Germ-free (GF) mice are more likely to produce T-helper type 2 (Th2) cells and IgE responses to dietary allergen than specific pathogen-free (SPF) mice (20), while the colonization of microbial population suppresses Th2 response and prevents mice from FA (21). The abundance of several bacterial families, including the *Lachnospiraceae, Lactobacillaceae, Rikenellaceae*, and *Porphyromonadaceae*, changed after ovalbumin (OVA) oral sensitization in food-allergy-susceptible mice (*Il4raF709* mice), while this change was not observed in sensitized WT mice (22). Different from the results of previous studies, a comparison of the microbiota composition of OVA allergic mice and non-responders found significantly higher abundances of sequence belonging to *Synthrophaceae* and *Ruminococcaceae* in non-responders (23).

The relationship between intestinal microbiota and food allergy strongly supported the critical role microbiota plays in the process of food allergy; however, the role of intestinal microbiota dysbiosis in food allergy and the mechanism of their interaction (24) need to be demonstrated. In the present study, we employed the C3H/HeJ mice and used OVA oral sensitization based on our previous protocol (25) to build a food allergy model. The high throughput sequencing of 16S rRNA was used to analyze differences in intestinal microbiota between allergic and negative control mice. And then the correlation of the changed intestinal bacterial flora and allergy-related immune cells in mesenteric lymph nodes (MLN) was also assessed. We found OVA sensitization without using an adjuvant induced allergic reactions and alteration of intestinal microbiota. The abundance of orders *Clostridiales* and *Mollicutes_RF39* were positively correlated to Tregs and dendritic cells (DCs) subgroup CD11C^+^CD103^+^ DCs in MLN of mice.

## Materials and Methods

### Animals and Experimental Protocols

All of the animal experiments in this research were performed according to the guidelines of the Institutional Animal Care and Use Committee. The animal experimental protocols were approved by the Experimental Animal Ethics Committee of the Capital Medical University with a license (AEEI-2018-128). In total, 24 specific pathogen-free (SPF) C3H/HeJ mice (female, 6 weeks old) with a body weight of 14.7–15.8 g were purchased from Vital River Laboratories (VRLs, Beijing, China) and bred and maintained in pathogen-free facilities in Capital Medical University. The mice were housed under conditions of 23°C/40–70% relative humidity and a 12h light/dark cycle with free access to a commercially available rodent diet and distilled water. Then the mice were randomly and evenly divided into two groups (OVA treated and Control group) with 12 mice per group and received different treatment after 1 week of acclimation. Four mice of the same group were set in one regular mouse cage.

**Figure 1:**
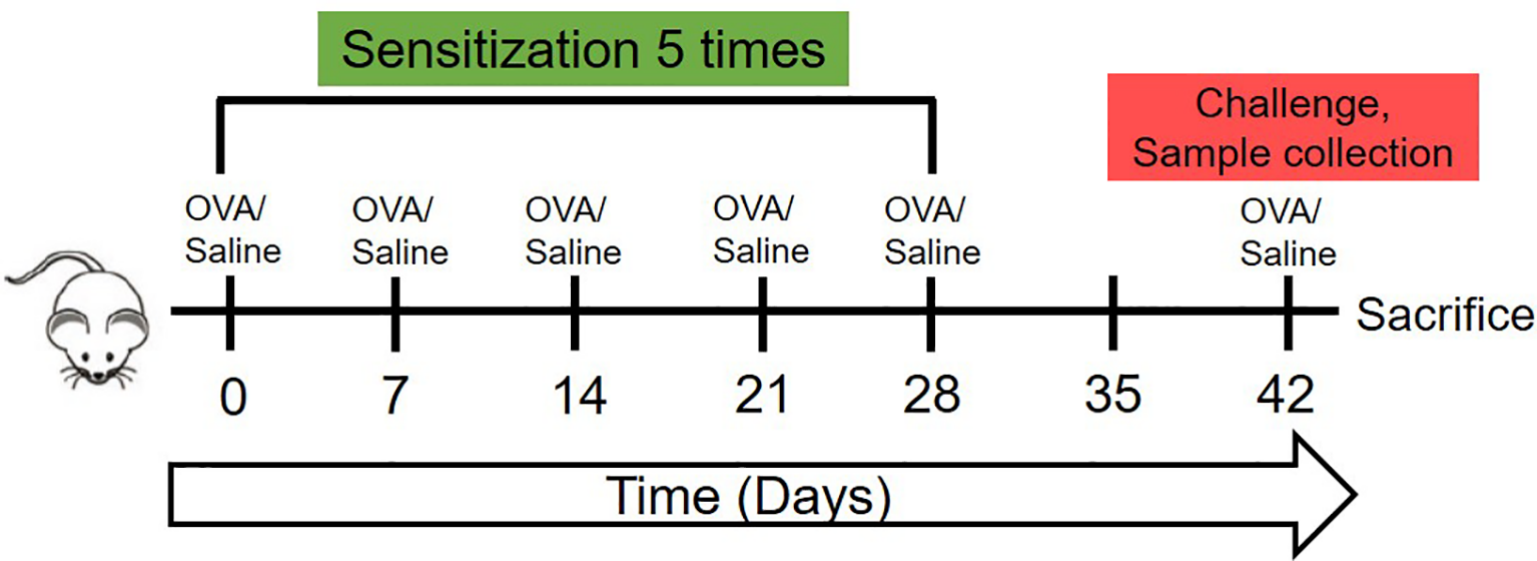
The schematic of the animal experiment of this research. The 7-week-old female C3H/HeJ mice were sensitized once weekly in the first 28 days with 1mg OVA dissolved in 200 ul saline or 200 ul saline by oral gavage. Then, on day 42, were orally challenged with 5mg OVA dissolved in 1 ml saline or 1 ml saline, followed by sera collection, temperature detection and sacrifice.

The experiment protocol is displayed in **Figure 1**. On days 0, 7, 14, 21, and 28 of the experiment, 200 uL pure saline or 200 uL saline containing 1mg OVA were gavaged to two groups of mice respectively for sensitization. On day 42, the challenge was achieved by intragastric administration of 1mL pure saline or 1 mL saline containing 5 mg OVA to mice in the two groups respectively. Blood samples were collected from the angular vein of each mouse 30 min after a challenge on day 42. Then the serum was collected, divided into four parts and stored in -80°C for batch-estimation of IgE, IgG1, IgG2a, and cytokines including Interferon-γ (IFN-γ), Interleukin-10 (IL-10), Interleukin-4 (IL-4), Transforming growth factor-β (TGF-β), and Mouse mast cell protease-1 (mMCP-1).

Besides, to estimate body temperature, the rectal temperature of each mouse was detected with a WI88375 probe (Beijing Science and Technology, Beijing) and recorded 10 min prior to the challenge and at 10 min intervals (for up to 50 min) after the challenge to assess their allergic reactions. Meanwhile, the symptoms of clinical anaphylaxis were monitored according to previous research (5): 0, no clinical symptom; 1, repetitive ear/mouth scratching and ear canal digging with hind legs; 2, decreased activity, self-isolation, and puffiness around eyes or mouth; 3, periods of motionless for more than 1 min, lying prone on the stomach, and decreased activity with an increased respiratory rate; 4, no response to whisker stimuli and reduced or no response to prodding; and 5, tremor, convulsion, and death. Then peritoneal lavage was performed with saline in each mouse and the saline was collected for detection of protein using a BCA kit (enhanced) purchased from Beyotime Biotechnology (Kit No. P0010S, Shanghai, China).

### Detection of Serum IgE, IgG1, IgG2a, Cytokines, and mMcp-1

The levels of OVA-specific IgE (sIgE), total IgG1, and IgG2a in serum of each mouse were tested using ELISA kits from Beijing Mecen Unicreate Bio-tech Co., LTD according to the protocols provided by the manufacturer. The concentration of IFN-γ, IL-10, IL-4, TGF-β, and mMcp-1 in the sera were measured using a multi-cytokine U-PLEX designed kit (Lot No. K152XWK-1 for TGF-β and K15069L-1 for others, Meso Scale Diagnoistics, LLC., Rockville, USA) according to the instruction of the manufacturer. The U-PLEX kit was designed to provide ultimate flexibility for the detection of several cytokines in only 25 ul sera through MSD electrochemiluminescence detection technology using SULFO-TAG_TM_ markers. Sensitivity levels of the kits were 0.1 μg/mL for IgE, 1.0 μg/mL for IgG1, 0.1 μg/mL for IgG2a, 0.16 pg/mL for IFN-γ, 3.8 pg/mL for IL-10, 0.56 pg/mL for IL-4, 37 pg/mL for TGF-β, and 1.4 pg/mL for mMcp-1.

### Measurement of Th1, Th2, Treg, and DCs Cells

The isolation of splenocytes and mesenteric lymph node (MLN) cells was performed as previously described (25). Then, the cells were divided into tubes with 510^6^ cells per tube. The FITC conjugated rat anti-mouse CD69, Brilliant Violet 421™ conjugated Armenian Hamster anti-mouse CD183 (CXCR3), and PE-conjugated rat anti-mouse IL-33Rα (ST2) monoclonal antibodies (mAb) were used to stain Th1 and Th2 cells. FITC conjugated rat anti-mouse CD4, PE-conjugated rat anti-mouse CD25, and APC-conjugated rat anti-mouse Foxp3 monoclonal antibodies (mAb) were used to stain Treg cells (Tregs). FITC-conjugated rat anti-mouse CD11C, PE-conjugated Armenian Hamster anti-mouse CD103, APC-conjugated rat anti-mouse CD80, PE/Cy7-conjugated rat anti-mouse CD86, and PerCP/Cyanine5.5-conjugated rat anti-mouse MHC class II (MHCII) mAbs were used to stain dendritic cells (DCs). The anti-mouse CD4, CD69, CD11C, and CD80 mAbs were purchased from Tonbo Bioscience (San Diego, CA, USA). The anti-mouse CD25 and Foxp3 mAbs were purchased from eBioscience (Waltham, MA USA). Additionally, the other mAbs were bought from BioLegend Inc (San Diego, CA, USA). The detailed staining steps were performed based on the previous description (26). Briefly, single-cell suspensions (510^6^ cells) were blocked with PBS containing 5% fetal calf serum (FCS) and 1% BSA, following with incubation of 30 min with either mAb. For detection of Foxp3, cells were permeabilized and incubated with anti-Foxp3 according to the manufacturer’s protocol (eBioscience). Finally, the stained cells were analyzed using a flow cytometer (NovoCyte, ACEA, Hangzhou, China). For each sample, more than 10000 events were required.

### Fecal DNA Extraction and PCR Amplification

To analyze the composition of intestinal microbiota, fecal samples of every mouse were collected on the 42nd day. To avoid contamination, two feces of each mouse were collected in an EP tube during defecation. Microbial community genomic DNA was extracted from 0.2–0.3g of feces using the E.Z.N.A.^®^ soil DNA Kit (Omega Biotek. Norcross, GA, U.S.) according to the manufacturer’s instructions. The DNA extract was checked on 1% agarose gel, and DNA concentration and purity were determined with NanoDrop 2000 UV-vis spectrophotometer (Thermo Scientific, Wilmington, USA). The hypervariable region V3-V4 of the bacterial 16S rRNA genes were amplified with primer pairs 338F (5’-ACTCCTACGGGAGGCAGCAG-3’) and 806R(5’-GGACTACHVGGGTWTCTAAT-3’) by an ABI GeneAmp^®^ 9700 PCR thermocycler (ABI, CA, USA), with an eight-base sequence barcode unique to each sample at the 5′ end of 338F and 806R, respectively. The PCR amplification of 16S rRNA gene was performed as follows: initial denaturation at 95°C for 3 min, followed by 27 cycles of denaturing at 95°C for 30 s, annealing at 55°C for 30 s and extension at 72°C for 45 s, and single extension at 72°C for 10 min, ending at 10°C. The PCR mixtures contain 4 μL of 5 × TransStart FastPfu buffer, 2 μL of 2.5 mM dNTPs, 0.8 μL of forward primer (5 μM), 0.8 μL of reverse primer (5 μM), 0.4 μL of TransStart FastPfu DNA Polymerase, 10 ng of template DNA, and finally up to 20 μL of ddH2O. The PCR product was extracted from 2% agarose gel and purified using the AxyPrep DNA Gel Extraction Kit (Axygen Biosciences, Union City, CA, USA) according to manufacturer’s instructions and quantified using QuantiFluor™ -ST (Promega, USA).

### Illumina MiSeq Sequencing

Purified amplicons were pooled in equimolar and paired-end sequenced (2 ×300) on an Illumina MiSeq PE300 platform (Illumina, San Diego, USA) according to the standard protocols by Majorbio Bio-Pharm Technology Co. Ltd. (Shanghai, China). The raw reads were deposited into the NCBI Sequence Read Archive (SRA) database (Accession Number: PRJNA658922).

### Bioinformatics Analysis

The raw 16S rRNA gene sequencing reads were demultiplexed, quality-filtered by Trimmomatic, and merged by FLASH with the following criteria: (i) the 300 bp reads were truncated at any site receiving an average quality score of <20 over a 50 bp sliding window, and the truncated reads shorter than 50 bp were discarded; (ii) exact barcode matching, 2 nucleotide mismatch in primer matching, and reads containing ambiguous characters were removed; and (iii) only overlapping sequences longer than 10 bp were assembled according to their overlapped sequence. Reads that could not be assembled were discarded. Then the number of obtained sequences was normalized by randomly subsampling each sample to the lowest level of sequences among samples.

Operational taxonomic units (OTUs) with 97% similarity cutoff (27) were clustered using UPARSE (version 7.1, http://drive5.com/uparse/), and chimeric sequences were identified and removed using UCHIME. The taxonomy of each OTU representative sequence was analyzed by RDP Classifier (http://rdp.cme.msu.edu/) against the 16S rRNA database (Silva SSU132) with a confidence threshold of 0.7.

The community richness, diversity, and evenness of species were assessed through several α-diversity indices, including Coverage, Ace, Chao, Shannon, Simpson, Phylogenetic diversity (PD), Shannoneven, and Sampsoneven, which were calculated using a mothur (version v.1.30.1) program basing on OTUs with 97% similarity. Wilcoxon rank-sum test was used to compare their diversity. The R package and Abund_jaccard distance metrix were used to perform Hierarchical clustering analysis on the OUT level. And the abund_jaccard distance metrix was also used to carry out Principal Component Analysis (PCA) and Principal coordinates analysis (PCoA) on the OUT level. Visualization of interactions among bacterial taxa in different samples was conducted using the R package. ANOSIM was performed to compare the microbiota composition between samples (28). Apart from the analysis above, Linear discriminant analysis (LDA) coupled with effect size measurements (LEfSe) analysis was conducted to search for statistically different bacteria species between two groups. Thus the different taxa from the phylum to the genus level were analyzed and visualized by taxonomic charts using the LEfSe tool (http://huttenhower.sph.harvard.edu/galaxy/root) since the analysis of the large number of OTUs detected in this study would be computationally too complex (29, 30).

### Statistical Analysis

All of the data were analyzed using SPSS 23.0 software and expressed as mean ± SD. GraphPad Prism V.5.0 (San Diego, CA, USA) was employed for graph preparation. The statistical analysis was performed using two-tailed Student’s test with the exception of 16S rRNA sequencing data, which were analyzed using a Wilcoxon rank-sum test. Spearman correlation analysis was performed for determining the correlation coefficient between distinguished intestinal flora and other allergy-related indicators. The *p* value of less than or equal to 0.05 was considered statistically significant. *p ≤0.05, **p ≤0.01, ***p ≤0.001.

## Results

### C3H/HeJ Mice Performed Significant Allergic Reactions to OVA

**Figure 2:**
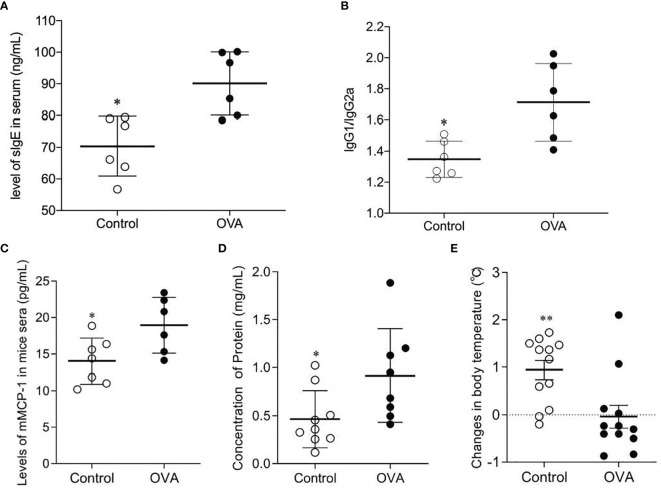
Concentrations of sIgE **(A)** and ratios of IgG1/IgG2a **(B)** in sera of OVA-treated (OVA) and saline-treated (Control) mice; Scatter plot of mMCP-1 level of mice serum **(C)**, protein concentration of peritoneal lavage fluid **(D)** and drop of temperature **(E)** in Control and OVA treated mice. The sera and peritoneal lavage fluid collection were conducted after the challenge, and protocols were described in *Methods*. The changes in body temperature were calculated by subtracting the rectal temperature (°C) of mice at 10 minutes before the challenge from the rectal temperature (°C) of mice at 50 minutes after the challenge. One plot denotes one sample, and the bar in the graph denotes Mean and SD. n=6-12/group. *p ≤ 0.05 vs OVA-treated group. Statistical analyses were performed with a two-tailed Student’s test. **p ≤ 0.01 vs OVA-treated group.

It has been verified in a previous experiment that the C3H/HeJ mouse did not show any specific immune responses to the commercially standard feeding. Thus, the concentrations of sIgE, IgG1, and IgG2a in the sera of mice were measured and the ratio of IgG1/IgG2a was calculated to estimate their allergic reaction to OVA through oral administration. As shown in **Figures 2A, B**, mice produced significantly higher levels of IgE in response to OVA oral treatment than did mice in Control group (*p ≤*0.05). And the ratios of IgG1/IgG2a in OVA treated mice were also significantly higher than those in Control mice (*p ≤*0.05). This suggested that the mice developed humoral immune responses to oral OVA sensitization.

To further verify the mice’s allergic reactions to OVA, concentrations of mMCP-1 in serum, levels of protein in peritoneal lavage fluid, drop in mice body temperature, and clinical anaphylaxis were measured. As shown in **Figure 2**, the sera of OVA-treated mice contained higher levels of mMCP-1, which is primarily released from mast cell degranulation, than those of Control mice (*p ≤*0.05, **Figure 2C**). The concentrations of protein in peritoneal lavage fluid of OVA treated hosts were remarkably higher than those in Control hosts (*p ≤*0.05, **Figure 2D**). In addition, the drop in body temperature in OVA-treated mice was also greater than that in Control mice (*p ≤*0.005, **Figure 2E**). Although there was no significant difference in anaphylaxis score between the two groups, OVA-treated mice showed higher scores when comparing with Control mice (**Figure S1**). These results suggested that the OVA-treated mice performed more severe mast cell degranulation, greater vascular permeability and more powerful allergic manifestation.

**Figure 3:**
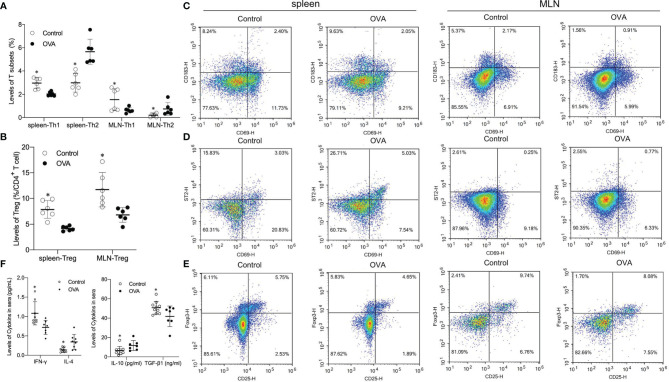
Splenic and MLN levels of Th1, Th2, Foxp3^+^ T regulatory (Treg) populations and concentrations of related cytokines in two groups. The bar in the graph denotes Mean and SD. n=6-12/group. *p ≤0.05 vs OVA-treated group; Statistical analyses were performed with two-tailed Student’s test. **(A)** Percentage of T subsets (Th1, Th2) in spleen and MLN. **(B)** Percentage of Treg in CD4+ T cell in spleen and MLN. **(C–E)** Representative images showing Th1, Th2 and Foxp3^+^ Treg levels in speen and MLN. **(F)** Level of cytokines (INF-γ, IL-4, IL-10 and TGF-β1) in sera of animals.

To determine whether the activation of Th1, Th2, and Treg cells underlies the observed allergic manifestations, levels of Th1, Th2, and Foxp3^+^ Treg cells in the spleen and MLN were detected. As shown in **Figure 3**, oral administration of OVA increased the proportion of effector Th2 cells in the spleen and MLN compared to saline treatment (*p ≤*0.05, **Figures 3A, D**). In contrast, the splenic and MLN levels of Th1 and Foxp3^+^ Treg cells in saline-treated hosts were significantly higher than those in OVA treated hosts (*p ≤*0.05, **Figures 3A–C, E**).

Different effector cells usually secrete different cytokines, which usually play different roles in the differentiation of cells. Thus concentrations of INF-γ, IL-4, IL-10, and TGF-β1 in the serum of mice were determined. Levels of INF-γ and TGF-β1, which are primarily related to differentiation and development of Th1 and Treg respectively, were significantly higher in the serum of Control mice than in OVA treated mice (*p ≤*0.05, **Figure 3F**). Levels of Th2 related cytokines IL-4 and IL-10 in sera collected from challenged OVA-treated mice were significantly higher than those from Control hosts (*p ≤*0.05, **Figures 3F, G**). These data suggested that the distinct allergic manifestations in OVA treated mice might be due to decreased levels of Th1 and Treg populations and increased proportions of Th2 subsets in the spleen and MLN.

### Relative Abundance of Matured Dendritic Cell Subsets in Mesenteric Lymph Nodes

**Figure 4:**
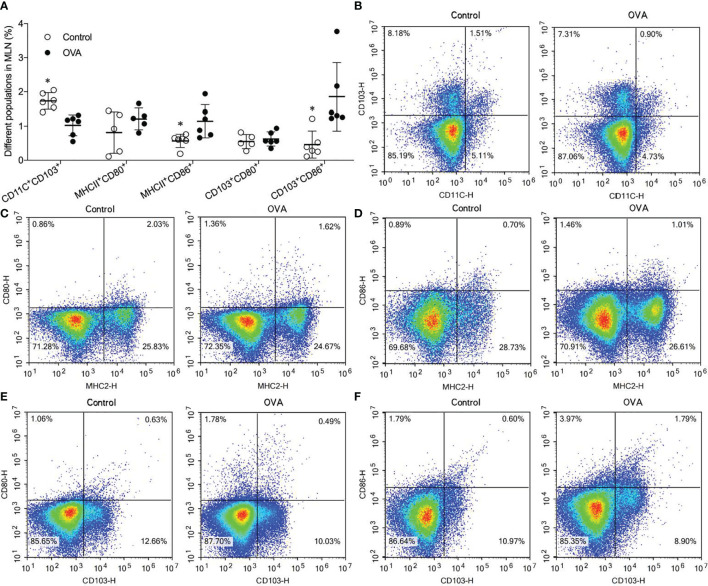
Levels of CD11^+^CD103^+^ DC, MHCII^+^CD80^+^ DC, MHCII^+^CD86^+^ DC, CD103^+^CD80^+^ DC, and CD103^+^CD86^+^ DC populations in the MLN of different treated mice. The bars in dot plot **(A)** indicate the percentages of five DCs populations with Mean + SD (n=6/group). The graph **(B–F)** appeals to the flow cytometry density maps of five DCs subsets in MLN of mice. *p ≤0.05 vs OVA-treated group; Statistical analyses were performed with two-tailed Student’s test.

During the development of food allergy, the activation of CD4^+^ T cells is usually affected by antigen present cells (APCs) (31). As an important APC, mature CD11^+^ DC in MLN plays a critical role in the activation and differentiation of Th1 and Th2 subsets through co-stimulation signal produced by CD80 or CD86 expressing on DCs (32). Furthermore, the levels of CD11^+^CD103^+^ DC, MHCII^+^CD80^+^ DC, MHCII^+^CD86^+^ DC, CD103^+^CD80^+^ DC, and CD103^+^CD86^+^ DC in the MLN and spleen of different treated mice were determined using flow cytometer. As shown in **Figure 4**, proportions of CD11^+^ DC in the MLN of saline-treated mice were significantly higher than those in OVA-treated mice (*p ≤*0.05, **Figures 4A, B**). In contrast, levels of MHCII^+^CD86^+^ DC and CD103^+^CD86^+^ DC were significantly lower in MLN of Control hosts than that in OVA treated hosts (*p ≤*0.05, **Figures 4A, D, F**). In addition, the levels of MHCII^+^CD80^+^ DC, and CD103^+^CD80^+^ DC did not show a remarkable difference between the two groups (*p* ≥0.05, **Figures 4A, C, E**). Although the levels of CD11C^+^ DC decreased in the MLN of allergic mice, proportions of MHCII^+^CD86^+^ DC and CD103^+^CD86^+^ DC, but not MHCII^+^CD80^+^ DC and CD103^+^CD80^+^ DC, increased during allergic reactions. In the spleen, most of the DC subsets did not show a noticeable difference between the two groups with the exception of CD11^+^CD103^+^ DC as shown in **Figure S2**.

### Microbiota Richness and Diversity Difference Between Two Groups

A total of 899552 usable sequence reads with a mean length of 416.73 ± 3.80 bp were obtained from 24 fecal samples, accounting for 79.28% of raw sequences according to demultiplexing and quality-filtering. Then, after subsampling each sample to an equal depth (21344 sequences per sample) and clustering, 694 operational taxonomic units (OTUs) at 97% identity were obtained, with OTUs ranging from 228 to 449 per sample. Coverage indices of all samples were more than 99%, indicating a sufficient sequencing depth in the two groups. The rarefaction analysis of species richness and Shannon indices based on OTUs level indicated that the data volume covered all species contained in fecal sample communities (**Figure S3**).

Analysis of alpha indices shown in **Table 1** compared the richness, diversity, and evenness of intestinal microbiota community in the two groups. The Chao and Ace estimators were significantly lower in OVA-allergic mice than in Control mice (*p ≤*0.0005), indicating that the microbial abundance in the stool of OVA-allergic mice was considerably lower. In addition, the Shannon, Simpson, and Phylogenetic diversity showed that the alpha-diversity was significantly lower in OVA-allergic hosts than in Control hosts (*p ≤*0.005). Furthermore, the extremely significant lower value of the Shannoneven and Simpsoneven indices (*p ≤*0.0005) in OVA-allergic mice demonstrated the considerably lower community evenness in feces of allergic mice. These results suggested that the richness, diversity, and evenness of the intestinal microbial community were all down regulated by OVA oral administration in mice.

**Table 1:** Comparison of α-indices of gut microbiota species in two groups.

Group	Richness estimator		Diversity indices			Evenness	
	Chao^***^	Ace^***^	Shannon^***^	Simpson^***^	PD^**^	Shannoneven^***^	Simpsoneven^***^
Control	461.93 ± 28.60	456.15 ± 23.76	4.51 ± 0.14	0.024 ± 0.0042	29.56 ± 1.28	0.751 ± 0.021	0.106 ± 0.018
OVA	351.50 ± 44.93	349.01 ± 40.13	3.70 ± 0.39	0.073 ± 0.044	25.38 ± 3.38	0.648 ± 0.058	0.057 ± 0.024

Values are expressed as Mean ± SD. n=12/group. **p ≤ 0.005 when Control vs OVA group, ***p ≤ 0.0005 when Control vs OVA group.

**Figure 5:**
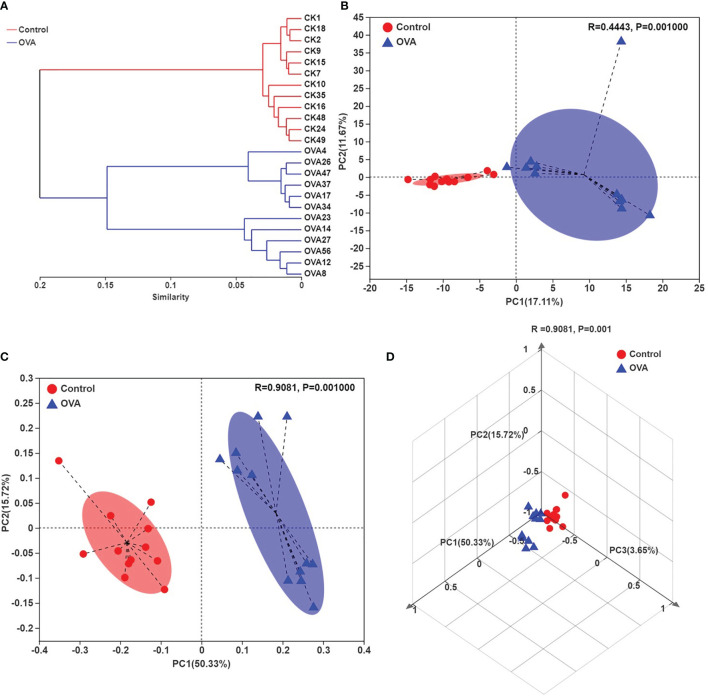
Hierarchical clustering tree on OUT level **(A)**, Principle component analysis (PCA) plots **(B)**, and principal coordinate analysis (PCoA) plots **(C, D)** on OUT level of all samples. **(C, D)** indicate the two- and three-dimensional PCoA plots.

The average hierarchical clustering analysis of β-diversity was conducted through abund_jaccard distances calculation to indicate the similarities among the fecal samples. As shown in **Figure 5A**, the samples from different treated mice can be clearly clustered into separate groups, although each group can be further divided into subgroups. Principle component analysis (PCA) revealed that the OVA treatment caused significant structural changes of fecal microbiota in mice (R =0.4443, *p* =0.001), as shown in plots (**Figure 5B**), and the PC1 and PC2 explained the 17.11% and 11.67% of variance respectively. Similarly, the principal coordinate analysis (PCoA) plots as shown in **Figures 5C, D** indicated that 50.33%, 15.72%, and 3.65% of the variation could be explained by PC1, PC2, and PC3 respectively with noticeable significance (R =0.9081, *p* =0.001). Both PCA and PCoA plots demonstrated that most samples from Control hosts had negative PC1 value while most samples from allergic animals had positive value. These results suggested significant different fecal microbiota community structures between the two treated groups.

### Community Structure of Stool Microbiota in Mice

**Figure 6:**
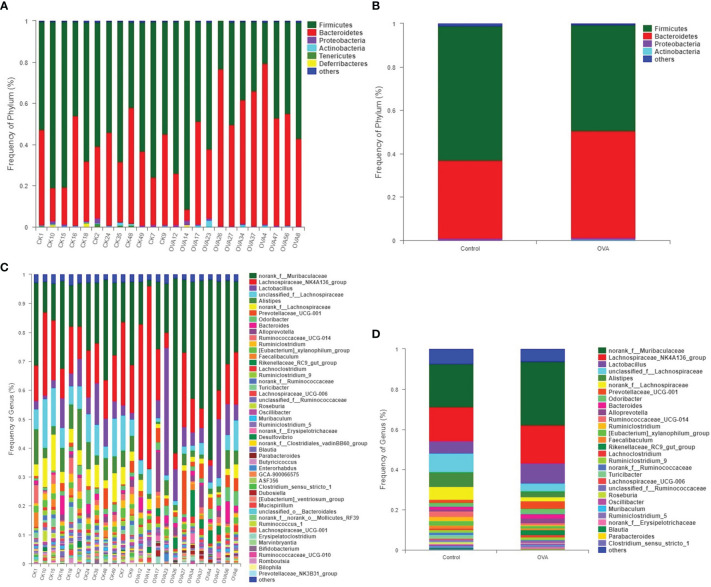
Distribution of fecal bacterial communities at the phyluam and genus levels for all mice. **(A, B)** show the phylum level; **(C, D)** show the genus level. **(A, C)** represent the intestinal flora composition of each sample, while **(B, D)** represent that of different groups.

Comparison of taxonomic composition in feces of different treated mice was conducted due to the distinguished difference in PCA and PCoA plots. The bar charts in **Figure 6** showed the distribution of taxonomic composition in each sample from two groups at the phylum and genus levels. There were four major phyla, including *Firmicutes*, *Bacteroidetes*, *Proteobacteria*, and *Actinobacteria* in all of the samples, as shown in **Figures 6A, B**. Among all the genera, a total of 29 genera were dominant, accounting for more 0.5% in all of the fecal samples, as shown in **Figures 6C, D**.

To further analyze the change of microbiota community in the intestinal tract of allergic mice, the significant differences in the relative abundance of fecal bacteria at different taxonomic levels were identified and presented in **Tables S1**–**S5**. Compared with those of the Control group, the relative proportions of phyla, including *Tenericutes*, *Deferribacteres*, and *Patescibacteria*, were significantly decreased in allergic mice (*p ≤*0.05, **Table S1**). And although the percentages of *Firmicutes* and *Bacteroidetes* did not show a significant difference between the two groups, the ratio of *Firmicutes/Bacteroidetes* decreased remarkably in allergic mice as shown in **Table S1** (*p ≤*0.05).

**Figure 7:**
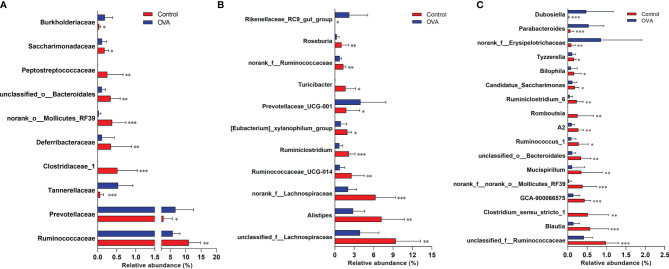
Comparisons of relative abundance of main bacterial populations at the family **(A)** and genus **(B, C)** levels. The bar charts indicate the percentages of bacteria with Mean + SD (n=12/group). *p ≤ 0.05; **p ≤ 0.01 and ***p ≤ 0.001 *vs* OVA-treated group; Statistical analyses were performed with Wilcoxon rank-sum test.

The different relative proportions of families and genera were presented in **Tables S4**, **S5**, and **Figure 7**. As shown in **Figure 7**, among the families with a relative abundance of more than 0.1%, 10 families were significantly different between mice in two groups. In comparison with the Control hosts, the relative abundances of *Ruminococcaceae*, *Clostridiaceae_1*, *Deferribacteraceae*, *norank_o:Mollicutes_RF39*, *unclassified_o:Bacteroidales*, *Peptostreptococcaceae*, and *Saccharimonadaceae* decreased significantly (*p ≤*0.05), while the proportions of *Prevotellaceae*, *Tannerellaceae*, and *Burkholderiaceae* increased significantly in the allergic hosts (*p ≤*0.05). At the genus level, 51 significantly different microbiota populations were identified between the two groups, as shown in **Table S5**. Among predominant genera accounting for more than 1% relative abundance in Control group, 9 genera, *unclassified_f:Lachnospiraceae*, *Alistipes*, *norank_f:Lachnospiraceae*, *Ruminococcaceae_UCG-014*, *Ruminiclostridium*, *[Eubacterium]_xylanophilum_group*, *Turicibacter*, *norank_f:Ruminococcaceae*, and *Roseburia* showed a significant decrease in allergic samples when compared with Control samples, while the other 2 genera of *Prevotellaceae_UCG-001* and *Rikenellaceae_RC9_gut_group* showed an increase (*p ≤*0.05, **Figure 7B**). Changes in the relative abundance of the other 17 less predominant genera, which account for 0.1% to 1% of fecal microbiota of mice, were also shown in **Figure 7C**. The different minor bacterial families and genera (relative frequency less than 0.1%) were not displayed in **Figure 7** due to the very low abundance levels of these bacteria which can’t explain biological significance strongly, and are presented in **Tables S4** and **S5**.

### Associations Between Fecal Microbiota Community and Allergy

To investigate the relationship of altered gut microbiota structure and food allergy, a Spearman correlation coefficient analysis between the relative abundance of noticeably changed genera and the subgroups of DCs, T cells in MLN, allergy-related cytokines, and symptoms was conducted based on the identification of statistically different bacteria species between two groups.

**Figure 8:**
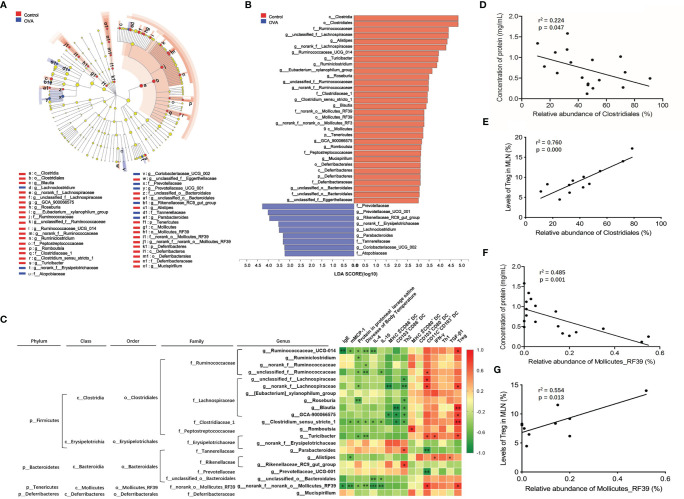
Identification of intestinal microbiota accounting for allergy and their correlation with allergic parameters in MLN. **(A)** Cladogram showing the phylogenetic distribution of the bacterial lineages associated with allergy from the two groups. Different-colored regions represent different constitutes. Circles indicate phylogenetic levels from phylum to genus. The diameter of each circle is proportional to the abundance of the group. **(B)** Indicator bacteria with LDA scores of 3 or greater in bacterial communities associated with allergy from two groups. **(C)** Heat map showing the Spearman correlation coefficient between identified significantly different species from phylum to genus level and the allergic indexes in MLN. **(D, E)** Spearman correlation of the order *Clostridiales* in intestinal microbiota with protein concentration in peritoneal lavage saline and Treg in MLN. **(F, G)** Spearman correlation of the order *Mollicutes_RF39* in intestinal microbiota with protein concentration in peritoneal lavage saline and Treg in MLN. *, ** and *** refer to significant Spearman correlation (*p ≤* 0.05, *p ≤* 0.01, *p ≤* 0.001).

Enriched species playing important roles in the differences between two groups were shown in cladograms, and LDA scores of three or more were confirmed by the LEfSe tool. According to **Figures 8A, B**, 18 genera of bacteria were significantly enriched in Control group (*p ≤*0.05). In OVA allergic group, six microbes on genus level were remarkably enriched, among which five genera were detected at a significant level (*p ≤*0.05) with the exception of *Lachnoclostridium* (*p* =0.053).

Among all of the identified significantly changed 23 genera, *unclassified_f:Eggerthellaceae* and *Coriobacteriaceae_UCG-002* accounted for less than 0.01% relative abundance. Therefore, the Spearman correlation coefficients of the remaining 21 genera and allergy-related parameters (IgE, mMCP-1, protein in the protoneal lavage saline, decrease in body temperature, IL-4, IL-10, IFN-γ, and TGF-β) of the cell subsets in MLN, referring to MHCII^+^CD86^+^ DC, CD103^+^CD86^+^ DC, Th2, MHCII^+^CD80^+^ DC, CD103^+^CD80^+^ DC, CD11C^+^CD103^+^ DC, Th1, and Treg, were analyzed, and the results were displayed in a heat map as shown in **Figure 8C**. Among all of the identified species, an abundance of genera *Ruminococcaceae_UCG-014, Ruminiclostridium, norank_f_Ruminococcaceae*, and *unclassified_f_Ruminococcaceae*, included in family *Ruminococcaceae*, were significantly positively correlated with Treg or CD11C^+^CD103^+^ DC in MLN and negatively correlated with protein in protoneal lavage saline and a decrease in body temperature, IgE, mMCP-1, IL-4, and IL-10 in serum. In family *Lachnospiraceae*, genera *unclassified_f:Lachnospiraceae, norank_f_Lachnospiraceae*, *Roseburia, Blautia*, and *GCA-900066575* were negatively related to protein in protoneal lavage saline, MHCII^+^CD86^+^ DC, CD103^+^CD86^+^ DC, and Th2 levels in MLN and positively related to Treg levels in MLN. The genus *Clostridium_sensu_stricto_1* belonging to family *Clostridiaceae_1* performed similar spearman correlation, negatively relating to protein in protoneal lavage saline, a decrease in body temperature, mMCP-1, IL-4, and IL-10 in serum, CD103^+^CD86^+^ DC and Th2 in MLN, and positively relating to Treg level in MLN. The abundance of genus *Romboutsia* belonging to the family *Peptostreptococcaceae* was positively correlated with levels of MHCII^+^CD80^+^ DC in MLN. An abundance of genus *Parabacteroides* belonging to family *Tannerellaceae* and genus *Rikenellaceae_RC9_gut_group* belonging to family *Rikenellaceae* was noticeably positively correlated to a proportion of Th2 in MLN. The level of genus *norank_f_norank_o_Mollicutes_RF39* in family *norank_o_Mollicutes_RF39* was negatively related to levels of IgE, mMCP-1, IL-4, and IL-10 in sera, protein in protoneal lavage saline, and a decrease in body temperature, while it was positively related to CD11C^+^CD103^+^ DC and Treg levels in MLN.

Besides, **Figure 8C** also displayed species from phylum to order containing statistically changed genera that were identified in OVA treated mice. Most of the genera positively relating to levels of Treg or MHCII^+^CD80^+^ DC or CD11C^+^CD103^+^ DC, while showing a negative correlation to Th2, Th2-related cytokines, MHCII^+^CD86^+^ DC, and CD103^+^CD86^+^ DC in MLN, belonged to orders *Clostridiales* and *Mollicutes_RF39*, classes *Clostridia* and *Mollicutes*, and phylum *Firmicutes* and *Tenericutes*. On the other hand, genera positively relating to Th2 subsets in MLN were included in order *Bacteroidales*, class *Bacteroidia*, and phylum *Bacteroidetes*. These results demonstrated that the gut microbiota community, especially families *Ruminococcaceae*, *Lachnospiraceae*, *Clostridiaceae_1*, and *norank_o_Mollicutes_RF39* play a critical role during the inhibition of food allergy, while the species belonging to families *Rikenellaceae* and *Tannerellaceae* prompted the development of food allergy. Furthermore, we also identified that orders of *Clostridiales* and *Mollicutes_RF39* were negatively related to protein concentration in protoneal lavage saline (r^2 =^ 0.224, *p* =0.047; r^2 =^ 0.485, *p* =0.001) and positively related to Treg in MLN (r^2 =^ 0.760, *p* =0.0002; r^2 =^ 0.554, *p* =0.0135) of mice, as shown in **Figures 8D–G**.

## Discussion

Although allergic responses between human and mice are substantially different (33, 34), mice models are commonly used to investigate the mechanism of allergic reactions (35, 36). The C3H/HeJ mice are a typical strain used in food allergy for their high susceptibility and remarkable hypersensitization reactions to food allergen (37, 38).

In this study, C3H/HeJ mice showed extensive reactions, such as increased IgE, IgG1, and mMCP-1, and Th2-associated cytokine levels, decreased Treg populations in immune tissues, as well as decreased body temperature after OVA treatment without adjuvant. In previous researches, similarly, the C3H/HeJ strain mice also displayed obvious humoral immune reactions, cellular immune responses, and systemic allergy symptoms to ovomucoid (OM) (39), peanut (40–42), and fish allergens (43) by oral administration combined with cholera toxin (CT). The route and mechanism of CT influencing the intestine might be the same as those by which some bacteria act on the intestinal barrier, although the CT is not involved in the pathogenesis of food allergy itself (44, 45). In addition to demonstrating that C3H/HeJ mice were sensitized successfully, it is worth noting that the mice herein also showed an altered intestinal microbiota after oral administration of OVA without using any adjuvant, suggesting that the OVA allergy might lead to changed intestinal flora. The disturbed intestinal flora described in previous researches, in turn, is considered one reason for food allergy since the use of CT corresponded to changes in intestinal microbiota (20, 46). Therefore, these studies suggest that gut flora and food allergy probably interact with each other rather than flora dysbiosis causing food allergy.

As the most potent antigen-presenting cells, the DCs, especially the CD11C^+^CD103^+^ DCs has been found to play a crucial role in the activation or regulation of T cells through inducing Foxp3^+^ Treg cells in the spleen and MLN (44, 47). According to the research of Fu et al., the maturation of CD11C^+^CD103^+^ DC induced regulatory T cells differentiation for the suppression of Th2-biased response (39). Thus, besides investigating the allergy-associated response, levels of CD11C^+^CD103^+^ DC as well as DC expressing mutation markers, including CD80, CD86, and MHCII in MLN, were also determined using flow cytometer in the current study. The decreased levels of CD11C^+^CD103^+^ DC and Tregs in the allergic group were consistent with those found in other studies (48), indicating that the CD11C^+^CD103^+^ DC may drive the differentiation of Tregs. Populations of CD103^+^CD86^+^ DC and MHCII^+^CD86^+^ DC increased in OVA allergic mice, while those of CD103^+^CD80^+^ DC and MHCII^+^CD80^+^ DC did not display a significant difference between the two groups. These results suggested an important role of CD86 expressing on DC in inducing food allergy. The study conducted by other researchers also reported similar results, showing that CD86 is an important factor in the induction of peanut allergy and the interaction between CD80 expression on DCs and CTLA-4 expression on T cells are both crucial for the induction of tolerance (49).

It is certain that the growing evidence points to an important role of the commensal microbiota in food allergy (24). We conducted a comparative structural analysis of intestinal microbiota from control and OVA-allergic mice. The major intestinal microbial community is composed of *Firmicutes*, *Bacteroidetes*, *Proteobacteria*, and *Actinobacteria*, which is consistent with the reported results of studies in mice (50) and human (51). The proportion of *Deferribacteres* was decreased in OVA-allergic mice compared with control hosts, which were dominated by *Deferribacteraceae*, which had decreased in OVA-treated mice. A previous report indicated that the level of *Deferribacteres* increased after oral treatment of beneficial probiotics in OVA-induced allergic mice (52). *Deferribacteres* in intestinal flora plays a role in the iron metabolism and iron balance of the gastrointestinal tract (53). It was inferred that food allergy is related to iron metabolism in the intestine. The richness of phylum *Tenericutes* also declined in allergic mice due to the decrease of bacterial family *norank_o_Mollicutes_RF39*. This is consistent with the result reported by Ogita T et al. (54), indicating an increased level of *Tenericutes* in combination with the reduction in the OVA allergic response. Studies have found that the phylum *Tenericutes* can digest fibers in the intestinal tract (55) and is conducive to maintaining the integrity of intestinal mucosa (56). So this result suggested that *Tenericutes* may associate with the protection of intestinal mucosa. Besides, similar to the results revealed in human research (57), the ratio of *Firmicutes/Bacteroidetes* also decreased in allergic mice, and this was attributed to the decreased proportion of *Firmicutes* and elevated richness of *Bacteroidetes* in OVA allergic hosts. However, there were also studies obtaining conflicting results with present research. Liu et al. reported increased richness of phylum *Deferribacteres* and ratio of *Firmicutes/Bacteroidetes* in mice with food allergy (50). We speculate this difference may be due to their use of adjuvants in OVA sensitization.

On the family level, we observed more than 10 altered intestinal flora in allergic animals. Belonging to *Clostridiales*, genera of *Ruminococcaceae, unclassified_o:Clostridiales*, and *Clostridiaceae_1* displayed a remarkable decrease in OVA allergic mice, and this is similar to previous research in human and mice (58–60). As a dominant bacterial community, *Ruminococcaceae* can decompose dietary fiber to produce short-chain fatty acids (SCFA) in the intestinal tract of mammals to protect intestinal mucosa (24, 61, 62). Studies indicated that *Clostridiales* plays an important role in intestinal homeostasis and in promoting Treg cell proliferation in intestinal immune tissues (63, 64). Thus, we infer that OVA sensitization downregulated the richness of order *Clostridiales* and destroyed its protection on intestinal mucosa while reduced the proliferation of Treg cells. Belonging to *Bacteroidetes*, the family *Tannerellaceae* showed an increased richness in allergic mice. This was consistent with results from a study in patients with Cohn’s disease (65) while its exact mechanism of influence on food allergy is unclear and needs to be investigated.

In recent years, it has been indicated that intestinal microbiota plays important role in immune homeostasis and the prevention of FA (22, 46, 64). The bacteriotherapy of FA has thus become a concern for many researchers. Esber and colleagues demonstrated the potentially beneficial role of three probiotic strains in cow’s milk allergy with regard to tolerance acquisition (66). Microbes from the *Clostridia* class and two other commensal species were enriched in healthy twins without FA (67). Abdel-Gadir and colleagues found that therapy with commensals including *Clostridiales* species suppressed FA in mice by inducing Treg cells expressing the transcription factor ROR-γt in a MyD88-dependent manner (68). In the present study, we confirmed the different crucial bacteria species between two groups using the LEfSe tool, on the basis of which the correlation of identified intestinal microbiota with levels of DC and T sub-populations in MLN were then analyzed. Most of the genera that were positively correlated with Treg and CD103^+^CD80^+^ DC (although without significance) belonged to *Mollicutes_RF39* and *Clostridiales*, which has been proved beneficial to intestinal immune balance through inducing Treg proliferation (64), protecting intestinal barrier function (69) and producing SCFA (5). In addition to previous research, considering the essential role played by intestinal DC subsets in the inhibition or prevention of food allergy (44) and the significant correlation with *Mollicutes_RF39* and *Clostridiales*, we may infer that OVA sensitization decreased the intestinal richness of *Mollicutes_RF39* and *Clostridiales*, which may induce Treg proliferation through CD103^+^CD80^+^ DC subsets in MLN. So *Mollicutes_RF39* and *Clostridiales* may be candidates in bacteriotherapy for FA in the future. This study may provide a reference for treating or preventing food allergy effectively by probiotic therapy. However, there are several limitations to this study. Firstly, although all of the mice were purchased from the same laboratory at the same time, the microbiota composites analysis should be conducted before OVA sensitization in order to demonstrate that the microbiota composites were altered by OVA treatment. Secondly, although OVA-specific Tregs has been demonstrated to suppress allergic reaction in previous research (70), they should be separated and used to treat OVA-sensitized mice to explore the exact mechanisms of OVA-induced food allergy without adjuvants in the present study.

## Conclusion

In conclusion, sensitization of C3H/HeJ mice using OVA without any adjuvant can induce remarkable allergic responses and noteworthy alteration of intestinal microbiota, demonstrating that allergen sensitization might cause disturbance of intestinal flora, which further confirms the relationship between food allergens, intestinal flora, and genetic susceptibility in the establishment of food allergy. This study also assessed the correlation between DC subsets, T subpopulation in MLN, and altered intestinal bacteria, which supplies novel possible evidence about the mechanism of *Mollicutes_RF39* and *Clostridiales* in promoting Treg proliferation. However, further studies using fecal microbiota transplantation (FMT) are needed to uncover the exact mechanism to provide persuasive evidence for the employment of *Mollicutes_RF39* and *Clostridiales* as anti-allergy probiotics.

## Data Availability Statement

The datasets presented in this study can be found in online repositories. The names of the repository/repositories and accession number(s) can be found below: https://www.ncbi.nlm.nih.gov/, PRJNA658922.

## Ethics Statement

The animal study was reviewed and approved by Experimental Animal Ethics Committee of the Capital Medical University.

## Author Contributions

CZ, W-WM, and RX conceived and designed the study. CZ carried out major experiments and collected data. CZ, L-LC, and R-QL performed the statistical analyses. CZ wrote the manuscript. W-WM and RX supervised the study. All authors contributed to the article and approved the submitted version.

## Funding

This work was supported by the Scientific Research Common Program of the Beijing Municipal Commission of Education (KM202010025002) and The National Natural Science Foundation of China (81502810).

## Conflict of Interest

The authors declare that the research was conducted in the absence of any commercial or financial relationships that could be construed as a potential conflict of interest.

## Supplementary Material

Supplementary Figure 1 Anaphylaxis score of animals in two groups. N=12.

Supplementary Figure 2 Levels of CD11+CD103+ DC, MHCII+CD80+ DC, MHCII+CD86+ DC, CD103+CD80+ DC and CD103+CD86+ DC populations in the spleen of different treated mice. The bars in dot plot **(A)** indicate the percentages of five DCs populations with Mean + SD (n=6/group). The graph **(B–F)** appeal the representative flow cytometry density images of five DCs subsets in spleen of mice. *, p ≤0.05 vs OVA-treated group; Statistical analyses were performed with two-tailed Student’s test.

Supplementary Figure 3 The Shannon indices based on OTUs level **(A)** and rarefaction analysis **(B)** of sequences in fecal samples.
