# Supplementary figures and images for: Alteration of Intestinal Microbiota Composition in Oral Sensitized C3H/HeJ Mice Is Associated With Changes in Dendritic Cells and T Cells in Mesenteric Lymph Nodes
Source: Front Immunol. 2021 Jun 10;12:631494. doi: 10.3389/fimmu.2021.631494 (PMC8222730; doi:10.3389/fimmu.2021.631494)

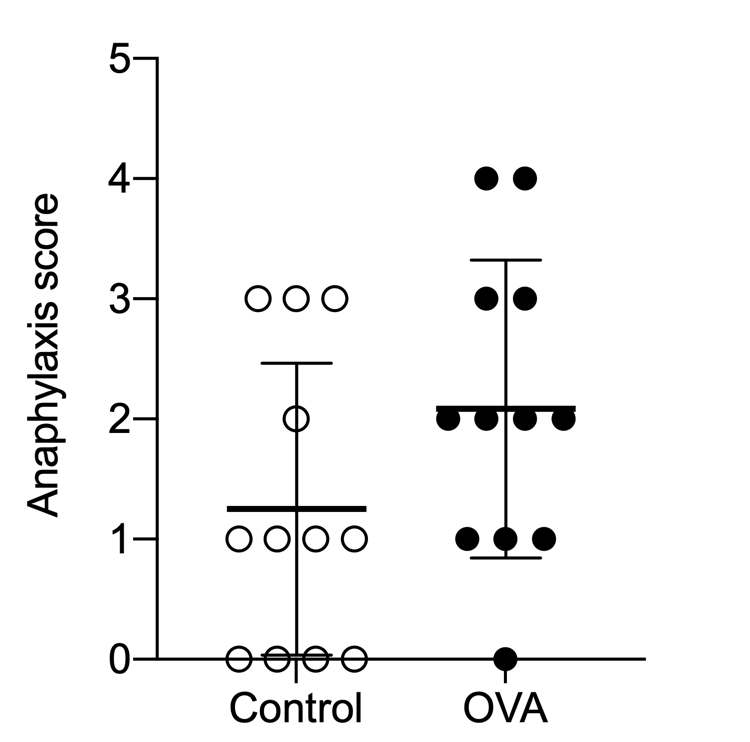

Supplement: Supplementary Figure 1 — Anaphylaxis score of animals in two groups. N=12. [file Image_1.tiff]

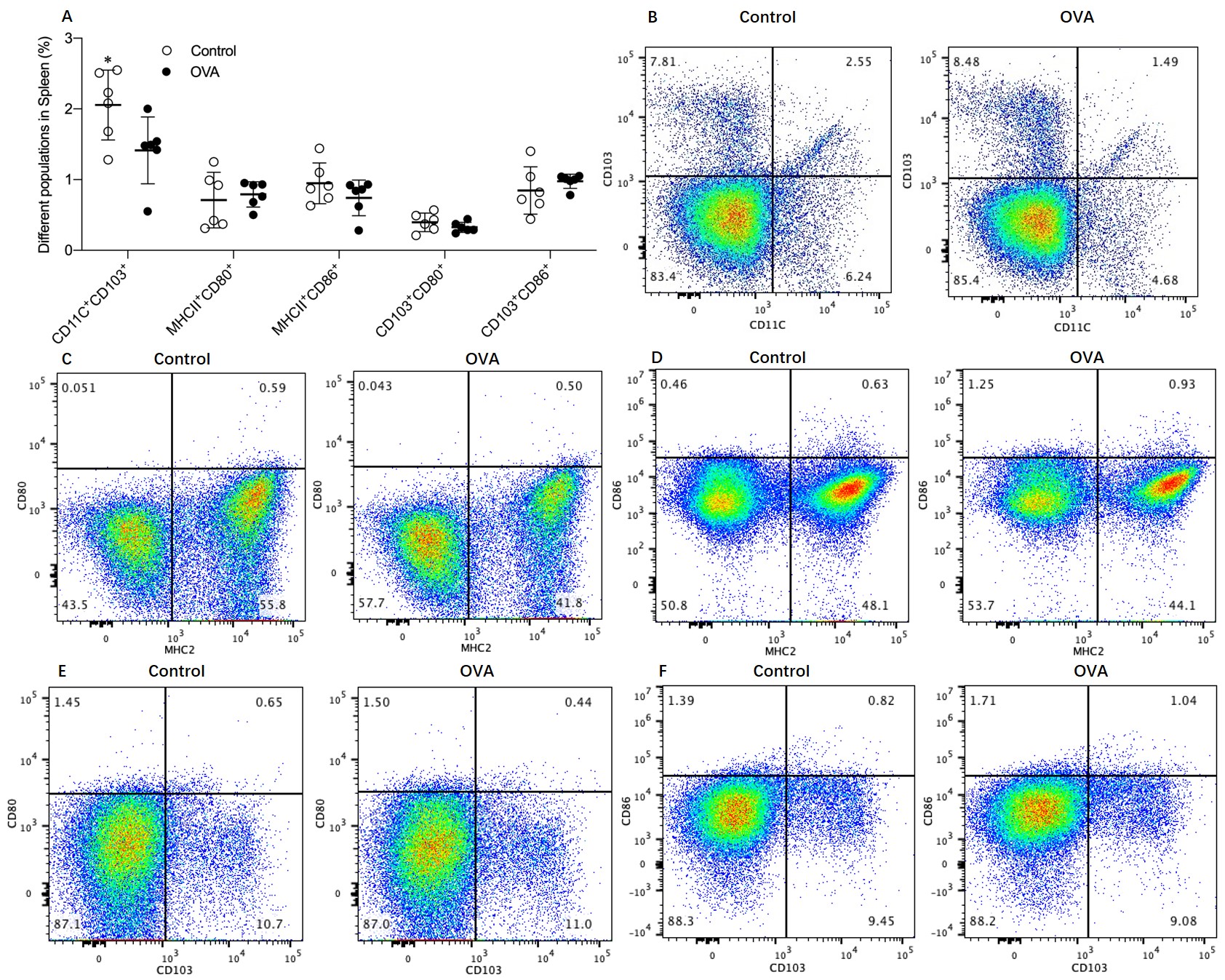

Supplement: Supplementary Figure 2 — Levels of CD11+CD103+ DC, MHCII+CD80+ DC, MHCII+CD86+ DC, CD103+CD80+ DC and CD103+CD86+ DC populations in the spleen of different treated mice. The bars in dot plot (A) indicate the percentages of five DCs populations with Mean + SD (n=6/group). The graph (B–F) appeal the representative flow cytometry density images of five DCs subsets in spleen of mice. *, p ≤0.05 vs OVA-treated group; Statistical analyses were performed with two-tailed Student’s test. [file Image_2.jpg]

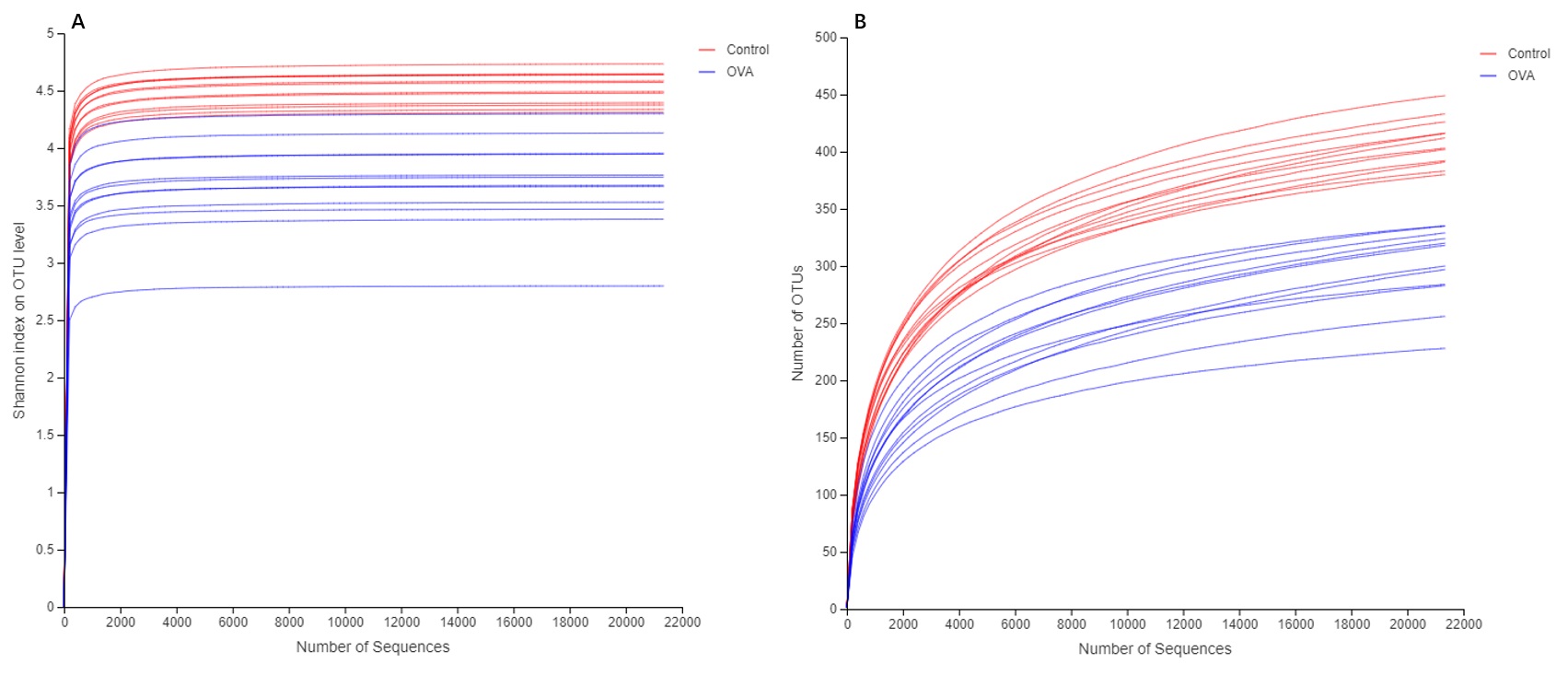

Supplement: Supplementary Figure 3 — The Shannon indices based on OTUs level (A) and rarefaction analysis (B) of sequences in fecal samples. [file Image_3.jpg]
