# Supplementary material for: Alteration of Intestinal Microbiota Composition in Oral Sensitized C3H/HeJ Mice Is Associated With Changes in Dendritic Cells and T Cells in Mesenteric Lymph Nodes
Source: Front Immunol. 2021 Jun 10;12:631494. doi: 10.3389/fimmu.2021.631494 (PMC8222730; doi:10.3389/fimmu.2021.631494)
Supplement: Supplementary file 4 [file DataSheet_1.docx]

Table S1. Percentage of the different fecal community phyla among Control mice and Allergic mice

| Group | Control | OVA |
| --- | --- | --- |
| *Tenericutes* | 0.39 ± 0.36** | 0.078 ± 0.066 |
| *Deferribacteres* | 0.35 ± 0.54** | 0.11 ± 0.32 |
| *Patescibacteria* | 0.18 ± 0.10* | 0.12 ± 0.12 |
| *Firmicutes/Bacteroidetes* ratio | 2.18 ± 1.41* | 1.05 ± 0.78 |

*, p≤0.05 when Control vs OVA group，**, p≤0.005 when Control vs OVA group.

Table S2. Percentage of the different fecal community classes among Control mice and Allergic mice

| Group | Control | OVA |
| --- | --- | --- |
| *Clostridia* | 52.36 ± 15.00* | 36.16 ± 24.66 |
| *Mollicutes* | 0.39 ± 0.36** | 0.078 ± 0.066 |
| *Deferribacteres* | 0.35 ± 0.54** | 0.11 ± 0.32 |
| *Saccharimonadia* | 0.18 ± 0.10* | 0.12 ± 0.12 |
| *Gammaproteobacteria* | 0.048 ± 0.042* | 0.20 ± 0.23 |

*, p≤0.05 when Control vs OVA group，**, p≤0.005 when Control vs OVA group.

Table S3. Percentage of the different fecal community orders among Control mice and Allergic mice

| Group | Control | OVA |
| --- | --- | --- |
| *Clostridiales* | 52.36 ± 15.00* | 36.16 ± 24.66 |
| *Mollicutes_RF39* | 0.38 ± 0.36*** | 0.03 ± 0.05 |
| *Deferribacterales* | 0.35 ± 0.54** | 0.11 ± 0.32 |
| *Saccharimonadales* | 0.18 ± 0.10* | 0.12 ± 0.12 |
| *Betaproteobacteriales* | 0.04 ± 0.04* | 0.19 ± 0.20 |
| *Anaeroplasmatales* | 0.01 ± 0.01** | 0.05 ± 0.05 |

*, p≤0.05 when Control vs OVA group; **, p≤0.005 when Control vs OVA group; ***, p≤0.0005 when Control vs OVA group.

Table S4. Percentage of the different fecal community Families among Control mice and Allergic mice

| Group | Control | OVA |
| --- | --- | --- |
| *Ruminococcaceae* | 10.96 ± 3.80** | 5.73 ± 2.35 |
| *Prevotellaceae* | 2.71 ± 2.88* | 6.66 ± 5.87 |
| *Clostridiaceae_1* | 0.52 ± 0.53*** | 0.00 ± 0.00 |
| *norank_o__Mollicutes_RF39* | 0.38 ± 0.36*** | 0.03 ± 0.05 |
| *Deferribacteraceae* | 0.35 ± 0.54** | 0.11 ± 0.32 |
| *unclassified_o__Bacteroidales* | 0.34 ± 0.25* | 0.11 ± 0.09 |
| *Peptostreptococcaceae* | 0.25 ± 0.41* | 0.00 ± 0.00 |
| *Saccharimonadaceae* | 0.18 ± 0.10* | 0.12 ± 0.12 |
| *Tannerellaceae* | 0.07 ± 0.09*** | 0.54 ± 0.39 |
| *Burkholderiaceae* | 0.04 ± 0.04* | 0.19 ± 0.20 |
| *Anaeroplasmataceae* | 0.01 ± 0.01** | 0.05 ± 0.05 |
| *Eubacteriaceae* | 0.00 ± 0.00* | 0.01 ± 0.01 |
| *Atopobiaceae* | 0.00 ± 0.00* | 0.002 ± 0.003 |

*, p≤0.05 when Control vs OVA group; **, p≤0.005 when Control vs OVA group; ***, p≤0.0005 when Control vs OVA group.

Table S5. Percentage of the different fecal community Genera among Control mice and Allergic mice

| Group | Control | OVA |
| --- | --- | --- |
| *unclassified_f__Lachnospiraceae* | 9.41 ± 3.83** | 3.87 ± 2.95 |
| *Alistipes* | 7.21 ± 3.52** | 2.83 ± 1.80 |
| *norank_f__Lachnospiraceae* | 6.28 ± 3.07*** | 2.05 ± 1.32 |
| *Ruminococcaceae_UCG-014* | 2.60 ± 1.95** | 0.78 ± 0.75 |
| *Ruminiclostridium* | 2.17 ± 0.95** | 0.70 ± 0.52 |
| *[Eubacterium]_xylanophilum_group* | 1.93 ± 0.68* | 0.92 ± 0.93 |
| *Prevotellaceae_UCG-001* | 1.76 ± 2.09* | 3.97 ± 3.92 |
| *Turicibacter* | 1.66 ± 1.54*** | 0.01 ± 0.02 |
| *norank_f__Ruminococcaceae* | 1.31 ± 0.38** | 0.74 ± 0.26 |
| *Roseburia* | 1.03 ± 1.06* | 0.28 ± 0.36 |
| *Rikenellaceae_RC9_gut_group* | 0.00±0.00* | 2.23±2.85 |
| *unclassified_f__Ruminococcaceae* | 0.99 ± 0.33*** | 0.41 ± 0.23 |
| *Blautia* | 0.57 ± 0.47** | 0.15 ± 0.15 |
| *Clostridium_sensu_stricto_1* | 0.52 ± 0.53*** | 0.00 ± 0.00 |
| *GCA-900066575* | 0.43 ± 0.16** | 0.14 ± 0.17 |
| *norank_f__norank_o__Mollicutes_RF39* | 0.38 ± 0.36*** | 0.03 ± 0.05 |
| *Mucispirillum* | 0.35±0.54** | 0.11±0.32 |
| *unclassified_o__Bacteroidales* | 0.34±0.25* | 0.11±0.09 |
| *Ruminococcus_1* | 0.28±0.25* | 0.09±0.12 |
| *A2* | 0.27±0.14* | 0.10±0.07 |
| *Romboutsia* | 0.25±0.41** | 0.00±0.00 |
| *Ruminiclostridium_6* | 0.23±0.17** | 0.05±0.08 |
| *Candidatus_Saccharimonas* | 0.18±0.10* | 0.12±0.12 |
| *Bilophila* | 0.16±0.19* | 0.08±0.17 |
| *Tyzzerella* | 0.16±0.06* | 0.11±0.09 |
| *Lachnospiraceae_FCS020_group* | 0.10±0.06* | 0.05±0.04 |
| *norank_f__Erysipelotrichaceae* | 0.09±0.10** | 0.85±1.06 |
| *Ruminococcaceae_UCG-005* | 0.09±0.04* | 0.04±0.04 |
| *Ruminococcaceae_NK4A214_group* | 0.08±0.05** | 0.03±0.02 |
| *Butyricimonas* | 0.07±0.06** | 0.02±0.03 |
| *Parabacteroides* | 0.07±0.09*** | 0.54±0.39 |
| *UBA1819* | 0.06±0.05*** | 0.00±0.00 |
| *Ruminococcaceae_UCG-013* | 0.06±0.04*** | 0.01±0.01 |
| *Tyzzerella_3* | 0.05±0.05*** | 0.00±0.00 |
| *Peptococcus* | 0.05±0.04* | 0.02±0.02 |
| *GCA-900066225* | 0.04±0.02*** | 0.01±0.01 |
| *Acetatifactor* | 0.03±0.03*** | 0.00±0.00 |
| *Anaerovorax* | 0.03±0.02*** | 0.00±0.00 |
| *norank_f__Desulfovibrionaceae* | 0.02±0.03* | 0.01±0.01 |
| *Negativibacillus* | 0.02±0.01*** | 0.00±0.00 |
| *Parasutterella* | 0.02±0.02* | 0.09±0.08 |
| *Dubosiella* | 0.02±0.02* | 0.48±0.70 |
| *Anaeroplasma* | 0.01±0.01** | 0.05±0.05 |
| *unclassified_f__Eggerthellaceae* | 0.01±0.01** | 0.00±0.00 |
| *Lachnospiraceae_AC2044_group* | 0.00±0.00** | 0.00±0.00 |
| *Anaerofustis* | 0.00±0.00* | 0.01±0.01 |
| *Rikenella* | 0.00±0.00* | 0.08±0.13 |
| *Coriobacteriaceae_UCG-002* | 0.00±0.00* | 0.00±0.00 |
| *[Eubacterium]_coprostanoligenes_group* | 0.00±0.00* | 0.00±0.01 |
| *Catabacter* | 0.00±0.00* | 0.01±0.02 |

*, p≤0.05 when Control vs OVA group; **, p≤0.005 when Control vs OVA group; ***, p≤0.0005 when Control vs OVA group.
